# Adverse Events of Massage Therapy in Pain-Related Conditions: A Systematic Review

**DOI:** 10.1155/2014/480956

**Published:** 2014-08-12

**Authors:** Ping Yin, Ningyang Gao, Junyi Wu, Gerhard Litscher, Shifen Xu

**Affiliations:** ^1^Acupuncture Department, Shanghai Municipal Hospital of Traditional Chinese Medicine, Shanghai 200071, China; ^2^Traumatology Department, Shuguang Hospital Affiliated to Shanghai University of Traditional Chinese Medicine, Shanghai 201203, China; ^3^Research Unit for Complementary and Integrative Laser Medicine, Research Unit of Biomedical Engineering in Anesthesia and Intensive Care Medicine, and TCM Research Center Graz, Medical University of Graz, 8036 Graz, Austria

## Abstract

Pain-related massage, important in traditional Eastern medicine, is increasingly used in the Western world. So the widening acceptance demands continual safety assessment. This review is an evaluation of the frequency and severity of adverse events (AEs) reported mainly for pain-related massage between 2003 and 2013. Relevant all-languages reports in 6 databases were identified and assessed by two coauthors. During the 11-year period, 40 reports of 138 AEs were associated with massage. Author, year of publication, country of occurrence, participant related (age, sex) or number of patients affected, the details of manual therapy, and clinician type were extracted. Disc herniation, soft tissue trauma, neurologic compromise, spinal cord injury, dissection of the vertebral arteries, and others were the main complications of massage. Spinal manipulation in massage has repeatedly been associated with serious AEs especially. Clearly, massage therapies are not totally devoid of risks. But the incidence of such events is low.

## 1. Introduction

Massage, as any systematic form of touch or manipulation performed on the soft tissues of the body to provide comfort and promote health [1–3], has become popular in the United States and the rest of the world in recent decades. It has also been recommended by the Chartered Society of Physiotherapy for the management of various pain-related conditions, especially those of musculoskeletal origin [4], such as neck pain, low back pain, headache, and migraine [5–8]. This is supported by numerous systematic reviews of a large number of randomized controlled trials (RCTs) [9–12]. Between 2002 and 2007, the 1-year prevalence of use of massage by the US adult population increased from 5% (10.05 million) to 8.3% (18.07 million), and massage belongs to one of the most popular complementary and alternative medicine (CAM) therapies in the USA [13]. The increased use brings attention to the safety and quality of the modality.

A number of large surveys on the safety of massage have been conducted. Most reported incidents have been fairly minor, and incidence rates were low. For example, from surveys and review articles, the risk of a serious irreversible complication (e.g., stroke) for cervical manipulations has been reported to vary from one adverse event in 3020 to one in 1,000,000 manipulations, and another review of the articles on complications of spinal manipulation, which identified 295 complications, yielded estimates of vertebrobasilar accidents from one in 20 000 patients to one per 1,000,000 cervical manipulations and cauda equina syndrome to be less than one per 1,000,000 treatments [14–16]. The authors of these studies concluded that serious AEs seem to be rare and massage is generally a safe intervention. So this systematic review seeks to evaluate all published data (between 2003 and 2013) about adverse effects of massage therapy. We specifically hope to help the clinician feel comfortable and informed in conversations with their patients regarding the appropriate, safe, and effective use of massage, not only in pain-related conditions.

## 2. Materials and Methods

### 2.1. Search Strategy

We searched 6 databases in an attempt to locate all existing case reports (irrespective of language of publication) with original data on AEs following any type of massage therapy published between January 2003 and June 2013 in electronic form. PubMed including MEDLINE, EMBASE, The Cochrane Library (via Wiley), CNKI, CQVIP, and Wanfang digital databases were searched. Search terms were “massage, manual therapy, tuina, and chiropractic.” These terms were combined with “safe, safety, adverse event, adverse reaction, side effects, complications, and risk.”

### 2.2. Inclusion and Exclusion Criteria

Only original case reports of complications or AEs of massage, manual therapy, and tuina published from January 2003 to June 2013 were included in this review. All those clinical study designs should be published in peer-reviewed journals, and like conference proceedings, cross-sectional and other descriptive designs and narrative reviews were excluded. Two coauthors independently screened the titles and abstracts of all papers found from the initial search. Disagreements between the two authors were resolved through discussion.

We excluded multiple inclusions and analyses of the same AEs as well as irrelevant studies. An irrelevant study was defined as a non-case report, such as a review, commentary, or clinical trial. Treatments not typically carried out by a massage therapist were also excluded, such as cardiac massage, prostatic massage, or carotid sinus massage. Adverse events related to massage oils, for example, allergies to aromatherapy oils or to the use of ice in conjunction with massage, were also excluded. All articles were evaluated and validated by one of the authors according to inclusion criteria.

### 2.3. Data Extraction

**Figure 1:**
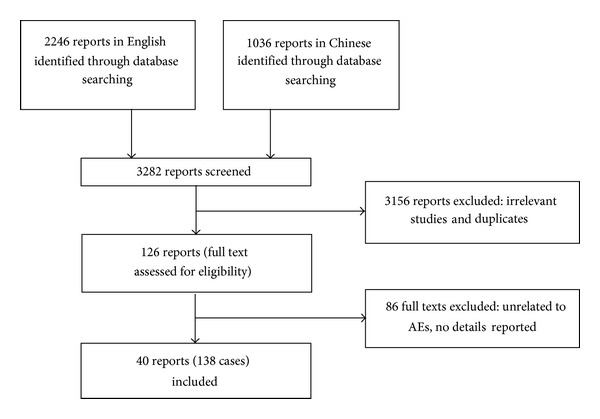
Flow chart of the screening process.

Electronic database searches identified a total of 3282 articles for consideration. After screening, 126 potentially relevant articles were identified for full review, and 40 studies met inclusion criteria finally. There were 86 articles that were excluded for being unrelated to AEs or for having no details reported (Figure 1). A full list of excluded articles is available from the corresponding author. When provided, we extracted author, year of publication, country of occurrence, participant related information (age, sex) or number of patients affected, the details of manual therapy, and clinician type that might have contributed to the AE, the reported AE, and its outcome. The data were extracted by two independent coauthors (P. Y. and NY. G.) and double checked to ensure matching and disagreements were resolved by consensus. Since there are no widely accepted criteria for judging the quality of AEs reports and the current studies' objective of describing case details, we did not assess the risk of bias on the included studies.

## 3. Results

The search strategy located 33 articles reporting a total of 43 case reports (in which the patients' age and/or sex were given) (Table 1), and a total of 7 reports containing 95 AEs in case series associated with massage were identified (Table 2). Most cases were reported from Asia especially in China (*n* = 24, 60% of total) and Europe (12, 30%), with few cases from the USA (3, 7.5%) and Australia (1, 2.5%), and more than half of the reported patients were female. There are 153 signs or symptoms of AEs in total, and the most common problems included disc herniation (25 cases, 16.3%), soft tissue trauma (17 cases, 11.1%), neurologic compromise (13 cases, 8.5%), spinal cord injury (13 cases, 8.5%), dissection of the vertebral arteries (10 cases, 6.5%), bone fracture (9 cases, 5.9%), hematoma or hemorrhagic cyst (6 cases, 3.9%), syncope (6 cases, 3.9%), cauda equina syndrome (4 cases, 2.6%), pain (2 cases, 1.3%), dislocation (2 cases, 1.3%), and others. The symptoms are frequently life-threatening, though in most cases the patient made a full recovery. In the majority of cases, the problems were related to spinal manipulations, including rotational movements, which seem to be the probable cause of the AEs.

## 4. Discussion

Our primary objective in reviewing the case reports of AEs associated with massage has been to identify individual cases and outbreaks of AEs then to analyze their possible causes, in order to minimize the massage AEs in future and enhance the practice safety within the profession. Of the 138 cases involving the AEs following massage in 40 references (Tables 1 and 2), spinal manipulation has repeatedly been reported with serious AEs especially. Collectively, these data suggest that massage is associated with frequent, mild, and transient AEs, but sometimes it may also be indeed associated with serious complications which can lead to permanent disability or even death. Although important details of most cases are poorly reported or frequently missing, these results have clear clinical and research related implications comparatively.

The true risk of injury due to spinal manipulation is still not known. Yet causal inferences may be not completely reasonable. Vascular accidents may happen spontaneously or could be caused by factors other than massage. The real serious incidence of AEs has been estimated to be ranging from 5 strokes in 100,000 manipulations to 1.46 case series in 10 million manipulations, and a rate of 2.68 deaths in 10 million manipulations has been reported [57–59]. The insurance industry claims [60] data support a risk of stroke as 1 per 2 million manipulations. 99% of all chiropractors practicing in Denmark completed a survey; they estimated that one case of cerebrovascular accident occurred for every 1.3 million cervical treatment sessions. The occurrence increased to 1 in every 900,000 treatment sessions for upper cervical manipulations, and they noted that techniques using rotational thrusts were overrepresented in the frequency of injury.

A temporal relationship is insufficient to establish causality, and recall bias can further obscure the truth. Moreover, denominators are rarely available. Smaller randomized controlled trials (RCTs) are unlikely to detect rare AEs, and better reporting of AEs is required, obviously. Therefore Senstad et al. [61–63] reported the data from 3 prospective investigations of 1778 adults who received chiropractic spinal manipulation indicated that 30% to 55% reported a minor adverse event. The most common were local discomfort (53% to 60%), radiating discomfort (10% to 23%), headache (10% to 12%), tiredness (11%), or nausea; dizziness, hot skin, or “other” reactions are uncommonly reported (<5% of reactions). And of the reported reactions, reactions were mild or moderate in 85% to 90% of patients. 64% of reactions appeared within 4 hours of treatment, and 74% to 83% had disappeared within 24 hours. Interestingly, reactions are most commonly reported by women and (for both genders) at the beginning of the treatment series. Patients with long-lasting problems are more likely to report treatment reactions, and patients with no prior experience of chiropractic care do not report more reactions than patients previously treated by chiropractors. Then Cagnie et al. [58] recruited 465 new patients treated with spinal manipulation by 59 physiotherapists (Belgian). All patients were asked to complete a questionnaire about AEs subsequently. 61% of the patients reported at least one AE, most of which were mild and transient, like headache (20%), stiffness (20%), local discomfort (15%), radiating discomfort (12%), and fatigue (12%). 61% of the problems had started within 4 hours after manipulation; 64% had resolved within 24 hours. No complications with long-lasting consequences were reported. Hurwitz et al. [64] reported the AEs documented in a 280-patient RCT which compared spinal manipulation with spinal mobilization as treatments for neck pain. 30% reported at least one AE. Patients receiving spinal manipulation were more likely to experience AEs than mobilization. The most frequently noted AEs were increase of pain, headache, tiredness, and radiating pain. 80% of the AEs began within 24 hours after treatment and were mild or of medium severity. No serious complications were noted. The three prospective case series above corroborate the results from several earlier studies [65] showing that mild to moderate AEs occur in a large proportion of patients receiving spinal manipulation, but these AEs are transient and nonserious. And recently, 767 patients were randomized to one of three treatment arms in a new study [66], to investigate differences in occurrence of adverse events between three different combinations of manual treatment techniques used by manual therapists (i.e., chiropractors, naprapaths, osteopaths, physicians, and physiotherapists) for patients seeking care for back and/or neck pain. And adverse events were measured with a questionnaire after each return visit and categorized into five levels. As a result, the most common adverse events were soreness in muscles, increased pain, and stiffness. The most frequent level of adverse event in this study was short minor lasting less than 24 hours and was rated less than or equal to three on the numeric rating scale regarding severity. No serious adverse events were reported.

Clearly, we should differentiate between various approaches. The above cases suggest that massage by nonprofessional and forceful techniques is often associated with AEs. In 8 cases the practitioners are massage therapists (5.8% of total) and 33 are chiropractors (23.9%), while in the other cases (70.3%) they are unregistered or even healthcare professionals only. So it might be unfair to assess the AEs of spinal manipulation as practiced by well-trained chiropractors alongside that associated with the untrained. Obviously from above, a variety of different care providers like physiotherapists, massage therapists, physicians, and osteopaths may perform a manipulation as part of their practice, but it should be most frequently performed by chiropractors [67]. Certainly skill and experience are important, and it is relevant to differentiate between different professions. But on the other hand, skill is a quality not easily controlled and some therapists are more skilled than others. Moreover, this review is aimed at evaluating the AEs of an intervention (massage) and not that of a profession (massage therapist/chiropractic). That is why in this review we show the implicated practitioners are not only chiropractors but also physicians, physiotherapists, “bonesetters,” and general medical practitioners.

This systematic review has several limitations. Even though the search strategy was deemed thorough, some relevant published articles might have been missed. It is possible that not all cases were identified in our searches. Although this paper has resulted in a few papers to review, it still had its strengths including the thorough search of the literature to help reduce bias in the review. We searched multiple relevant electronic databases and used two coauthors to determine articles for inclusion in the review and to evaluate the literature. But because of the inherent nature of case reports and other anecdotal reports, it is impossible to make inferences regarding cause and effect. Therefore, it is not known whether the serious AEs in cases identified in this review were caused by massage and whether the association between therapy and event was accidental or not. So the safety in massage is still far from being achieved. Further investigations are urgent to assess definite conclusions regarding this issue. In the meantime, it should be necessary to establish a system of risk alert for guaranteed surveillance on this type of CAM and safe practice guidelines are required and could continue to be enforced.

## 5. Conclusions

In conclusion, although serious AEs associated with massage in general and pain-related massage in particular are few, massage therapies are not totally devoid of risks. Spinal manipulation in massage has repeatedly been associated with serious AEs especially. But the incidence of such events is probably low. Adequate regulation could further minimize the risks. So we recommend that not only adequate training in biomedical knowledge for practitioners, such as anatomy and microbiology, but also safe practice guidelines are required and should continue to be enforced in order to minimize massage AEs.

## Figures and Tables

**Table 1:** Cases of AEs associated with massage therapy.

Author (year)	Country	Language	Age, sex	Details of manual therapy	Clinician type	Adverse event (nature and location)	Follow-up
Jay et al. (2003) [17]	USA	English	26, F	Chiropractic manipulations	Chiropractor	Bilateral dissection of vertebral arteries followed by bilateral occipital-parietal hemorrhagic infarction and visual impairment	Complete resolution (20 d.)

Beck et al. (2003) [18]	Germany	English	40, F	Axial tension and rotation	Chiropractor	Intracranial hypotension	Complete resolution confirmed by MRI

Nadgir et al. (2003) [19]	USA	English	34, M	Neck manipulation	Chiropractor	Neck cramping (bilateral internal carotid and vertebral artery dissection)	Minimal residual hemianesthesia and dysesthesia

Oehler et al. (2003) [20]	Germany	German	31, F	Chiropractic neck manipulation	Unknown	Bilateral dissections of vertebral arteries	Resolution

Yokota et al. (2003) [21]	Japan	Japanese	38, M	Chiropractic neck manipulation	Unregistered practitioner	Dissection of left vertebral artery followed by Dejerine syndrome	Unknown

Licht et al. (2003) [22]	Denmark	English	39, M	Cervical manipulation	General practitioner	Large infarction in the left cerebellar hemisphere (presumably due to arterial dissection)	Complete recovery (3 mo.)

Xiong (2003) [23]	China	Chinese	39, M	Reduction manipulation	Not mentioned	Cerebral infarction	Irritating cough and limb numbness (2 y.)

Ma and Xu (2003) [24]	China	Chinese	50, F	Rotation	Not mentioned	Peripheral nerve entrapment syndrome	Recovered

Yu et al. (2003) [25]	China	Chinese	42, M	Manipulative reduction	Not mentioned	Spinal cord injury	Recovered (6 mo.)

Yu et al. (2003) [25]	China	Chinese	22, M	Manipulative reduction	Not mentioned	Spinal cord injury	Symptom remission (15 d.)

Zhang et al. (2003) [26]	China	Chinese	35, M	Rotation	Not mentioned	Extrusion of lumbar intervertebral discs (lower limb pain, incontinence, and saddle sensation disorders)	Pain relief after surgery, but residual saddle area numbness

Zhang et al. (2003) [26]	China	Chinese	48, M	Rotation	Not mentioned	Extrusion of lumbar intervertebral discs (lower limb pain, walking and sexual dysfunction)	Muscle recovery after surgery, but still sexual dysfunction (1 y.)

Izquierdo-Casas et al. (2004) [27]	Spain	Spanish	37, F	Chiropractic	Not mentioned	Dissection of vertebral artery followed by tetraparesis	Locked-in syndrome

Morandi et al. (2004) [28]	France	English	49, F	Lumbar vertebral manipulation	Physician	Caudal spinal cord ischemia	Permanent neuroloss

Saxler and Barden (2004) [29]	Germany	German	27, F	Cervical chiropractic manipulation (C5/6), facet joint infiltration	Not mentioned	Epidural hematoma extending from cervical to sacral spine	Complete resolution

Tomé et al. (2004) [30]	Spain	Spanish	Not noted	Chiropractic manipulation	Not mentioned	Multiple cervical disc herniation	Not mentioned

Hansis et al. (2004) [31]	Germany	German	45, M	Chiropractic manipulation	Unknown	L4 fracture osteoporosis	Surgery

Hansis et al. (2004) [31]	Germany	German	38, M	Unknown	Unknown	Disk protrusion	Surgery

Wang et al. (2004) [32]	Australia	English	82, F	Lumbosacral manipulation	Unknown	Extradural hemorrhagic synovial cyst, leg pain	Complete recovery after L3–L5 laminectomy and cyst removal

Wang et al. (2004) [32]	Australia	English	76, F	Lumbosacral manipulation	Unknown	Hemorrhagic synovial cyst with resultant lumbar canal stenosis and exacerbation of severe pain in buttock and left leg pain	L4-L5 laminectomy and cyst removal with excellent outcome

L. Zhang and G. H. Zhang (2004) [33]	China	Chinese	15, F	Rotation	Not mentioned	Atlantoaxial dislocation	Recovered after surgery (2 wk.)

Chen et al. (2005) [34]	Taiwan	English	72, M	Chiropractic and massage therapy	Not mentioned	Neck pain, relieved by chiropractor, hematoma of ligamentum flavum at the level of C3-C4 with hemiparesis	Complete recovery after laminectomy (1 y.)

Suh et al. (2005) [35]	Korea	English	37, F	Axial tension and rotation	Chiropractor	Intracranial hypotension	Complete resolution after epidural blood patch

Schmitz et al. (2005) [36]	Germany	English	37, F	Cervical manipulation	General medical practitioner	Displaced odontoid fracture in the presence of an aneurismal bone cyst	Complete recovery after surgery

Chen et al. (2005) [37]	China	Chinese	48, F	Rotation	Self-treatment by her husband	Cervical myelopathy (neck pain, dizziness, and numbness of limbs)	Recovered (28 d.)

Jing and Yang (2006) [38]	China	Chinese	41, M	Rotation	Not mentioned	Fracture and bulge of intervertebral discs	Nearly full recovery after surgery

Solheim et al. (2007) [39]	Norway	English	77, M	Lumbar manipulation therapy	Chiropractor	Partial cauda equina syndrome due to spinal epidural hematoma in the L3 region	Surgical evacuation of hematoma via L3 and L4 laminectomies, improvement with motor deficits, but the bladder dysfunction remained

Guo et al. (2007) [40]	China	Chinese	78, F	Lumbar manipulation therapy	Not mentioned	Rib fracture (the seventh rib)	Not mentioned

Guo et al. (2007) [40]	China	Chinese	60, M	Cervical manipulation	Not mentioned	Lacerations of soft tissues	Recovered (1 mo.)

Guo et al. (2007) [40]	China	Chinese	48, M	Lumbar manipulation therapy	Not mentioned	Fracture (L3 transverse process fractures)	Not mentioned

Guo et al. (2007) [40]	China	Chinese	67, F	Lumbar manipulation therapy	Not mentioned	Fracture (L2 transverse process fractures)	Not mentioned

Guo et al. (2007) [40]	China	Chinese	49, F	Cervical manipulation	Not mentioned	Syncope	Not mentioned

Guo et al. (2007) [40]	China	Chinese	53, M	Rotation	Not mentioned	Fracture (proximal humeral fracture)	Not mentioned

Yi et al. (2008) [41]	China	Chinese	45, F	Cervical manipulation	Not mentioned	Hypochondriacal neurosis	Not mentioned

Yi et al. (2008) [41]	China	Chinese	54, F	Cervical spine manipulative reduction	Not mentioned	Hypochondriacal neurosis	Recovered

Jiang (2008) [42]	China	Chinese	28, M	Rotation	Massage therapist (private clinics)	Brown-Sequard syndrome due to spinal epidural hematoma	Near full recovery after surgery (3 wk.)

Huang et al. (2010) [43]	Taiwan	English	51, M	Manipulation directed at the lumbopelvic-thigh region and massage	Physiotherapist	Rupture of soft tissue tumor at anterior proximal thigh	Surgical tumor resection, and neither recurrence nor metastasis was observed 48 months after surgery

Zhu (2010) [44]	China	Chinese	35, F	Joint mobilization	Not mentioned	Hemarthrosis of knee joint	Improved the joint activity (4 mo.)

Jin et al. (2010) [45]	China	Chinese	46, not noted	Rotation	Massage therapist	Dead	Dead

Tamburrelli et al. (2011) [46]	Italy	English	42, M	Spinal manipulation	Doctor of chiropractic	Cauda equina syndrome, L5-S1 extrusion	L5 laminotomy and L5-S1 discectomy, improved, but with persistent bowel dysfunction, impotence, lower extremity; pain, paresthesias, and mild sensory deficit

Bi (2011) [47]	China	Chinese	59, M	Cervical manipulation	Not mentioned	Dorsolateral medullary syndrome	Improved (14 d.)

Zhang et al. (2011) [48]	China	Chinese	29, F	Rotation	Massage doctor	Atlantoaxial dislocation	Near full recovery after surgery (3 mo.)

Li et al. (2012) [49]	China	Chinese	37, F	Neck massage	Not mentioned	Vertebral arterial dissecting aneurysm	Horner syndrome disappeared and without dysphagia (3 mo.)

**Table 2:** Case series of AEs associated with massage therapy.

Author (year)	Country	Language	Cases	Details of manual therapy	Clinician type	Adverse event (nature and location)	Follow-up
Young and Chen (2003) [50]	Taiwan	English	9	Cervical manipulation	Chiropractor	Vertebral artery occlusion (1 case); stenosis (1 case); slow blood flow (1 case) associated with normal findings (6 cases)	Recovered (3 mo.)

Mei et al. (2003) [51]	China	Chinese	21	Rotatory reduction manipulation	Not mentioned	Nausea and profuse sweating (8 cases); headache and vertigo (5 cases); upper extremity numbness (4 cases); cervical limitation of activity (12 cases); lower limbs motor disturbance (5 cases)	8 cases recovered, 13 cases improved

Oppenheim et al. (2005) [52]	USA	English	18	Spinal manipulation	Chiropractor	Spinal cord injuries (9 cases); cauda equina syndrome (2 cases); radiculopathy (6 cases); pathological fracture (3 cases)	16 patients need surgery, but half of them made an excellent recovery subsequently, and one-third had a good recovery

Wang (2005) [53]	China	Chinese	9	Rotatory reduction manipulation	Not mentioned	Lumbar intervertebral disc extrusion	Fully recovered (5 cases); foot prolapse (3 cases); hypoesthesia (1 case)

Wang et al. (2008) [54]	China	Chinese	5	Neck massage	Not mentioned	Cervical disc herniation	Recovered

Guo and Lu (2009) [55]	China	Chinese	26	Rotation (17 cases), tendon-regulating method (9 cases)	Not mentioned	Simple soft tissue injury (15 cases); cervical structural damage (11 cases)	Not mentioned

Qu et al. (2010) [56]	China	Chinese	7	Pressing manipulation	Not mentioned	Aggravated lumbar intervertebral disc extrusion	Recovered (5–10 d.)
